# Ondansetron treatment reduces rotavirus symptoms—A randomized double-blinded placebo-controlled trial

**DOI:** 10.1371/journal.pone.0186824

**Published:** 2017-10-27

**Authors:** Marie Hagbom, Daniel Novak, Malin Ekström, Younis Khalid, Maria Andersson, Magnus Lindh, Johan Nordgren, Lennart Svensson

**Affiliations:** 1 Department of Clinical and Experimental Medicine, Division of Molecular Virology, Medical Faculty, Linköping University, Linköping, Sweden; 2 Sahlgrenska University Hospital, The Queen Silvia Children’s Hospital, The Emergency Department, Gothenburg, Sweden; 3 Department of Infectious Diseases/Section of Clinical Virology, Institute of Biomedicine, University of Gothenburg, Gothenburg, Sweden; 4 Department of Medicine, Karolinska Institute, Stockholm, Sweden; University Medical Center Goettingen, GERMANY

## Abstract

**Background:**

Rotavirus and norovirus cause acute gastroenteritis with severe diarrhoea and vomiting, symptoms that may lead to severe dehydration and death. The objective of this randomized double-blinded placebo-controlled study was to investigate whether ondansetron, a serotonin receptor antagonist could attenuate rotavirus- and norovirus-induced vomiting and diarrhoea, which would facilitate oral rehydration and possibly accelerate recovery and reduce need for hospitalization.

**Methods:**

Children with acute gastroenteritis, aged 6 months to 16 years where enrolled (n = 104) and randomized to one single oral dose (0.15mg/kg) of ondansetron (n = 52) or placebo (n = 52). The number of diarrhoea and vomiting episodes during the 24 hours following treatment was reported as well as the number of days with symptoms. Pathogens in faeces were diagnosed by real-time PCR. Outcome parameters were analyzed for rotavirus- and norovirus-positive children.

**Results:**

One dose of oral ondansetron reduced duration of rotavirus clinical symptoms (p = 0.014), with a median of two days. Furthermore, ondansetron reduced diarrhea episodes, most pronounced in children that had been sick for more than 3 days before treatment (p = 0.028).

**Conclusion:**

Ondansetron may be a beneficial treatment for children with rotavirus gastroenteritis.

**Trial registration:**

European Clinical Trial Database EudraCT 2011-005700-15.

## Introduction

Diarrhoeal disease is the second cause of death in children under 5 years of age, causing approximately 760 000 deaths each year[1]. Rotavirus (RV) is the major cause of these deaths, with norovirus (NoV) being the second most predominant viral pathogen. Rotavirus infections accounts for about a third of severe cases of acute gastroenteritis (AGE) requiring hospitalisation[2], and 200 000 children die annually due to RV infection, mainly in developing countries[2, 3].

First-line treatment of AGE focuses on recovering the fluid balance by oral rehydration therapy (ORT). However, extensive vomiting complicate ORT treatment, and in children with severe dehydration paediatricians are more likely to choose intravenous administration instead of ORT[4]. Antiemetic drugs such as granisetron and ondansetron (serotonin-3 (5-hydroxytryptamine-3, 5-HT_3_) receptor antagonists), have been shown to have a good antiemetic effect in patients receiving chemotherapy and in situations with vomiting after surgery[5, 6]. Furthermore, ondansetron was shown to facilitate ORT in children with AGE in independent clinical studies[7–13], although etiologies were only investigated in one of the studies and pathogens were not taken into account for the outcome result[12].

Ondansetron is one of the best known 5-HT_3_ receptor antagonists, blocking receptors at vagal and sympathetic nerves and the chemoreceptor triggering zone[14]. However, 5-HT_3_ receptor antagonists not only inhibit transmission of signals to the CNS, they also decrease intestinal motility, presumably by interfering with serotonergic neurotransmission within the enteric nervous system (ENS) and blocking the initiation of reflexes[15].

Although ondansetron has been shown to have effect on the reduction of vomiting during AGE, it is not yet known whether vomiting and diarrhoea specifically caused by RV and NoV can be attenuated with ondansetron in humans.

We have previously shown that RV infection activates the enteric nervous system (ENS) [16–18], stimulate release of serotonin (5-hydroxytryptamine, 5-HT) from enterochromaffin (EC) cells and activates brain structures in the vomiting centre[19]. Recently we also demonstrated that ondansetron can attenuate RV-induced diarrhoea in mice and that treated infected mice gained more weight and shed less virus[20]. These observations, together with the fact that 5-HT_3_ receptor antagonists have constipation as a side effect[15, 21] and are used as a treatment in irritable bowel syndrome (IBS) dominated by diarrhoea[22, 23], led us to test the hypothesis whether ondansetron could reduce vomiting and diarrhoea in children with RV or NoV infection.

## Methods

### Ethics approval

This trial (S1 Study Protocol) was approved by the ethics committee of the Linköping University in Sweden (Dnr: 2011/502-31, 2013/27-32, 2014/288-32) and by the Medical Products Agency (MPA, Uppsala, Sweden) and conducted according to the Good Clinical Practice guidelines. The study is registered at the European clinical trial database EudraCT (2011-005700-15). Date of registration: 2011-12-28.

### Study population

Children aged between 6 months and 16 years, with vomiting and diarrhoea were recruited in the children´s emergency department at the Queen Silvia Children's Hospital Gothenburg Sweden. Children and/or their parents recived written information about the study and were informed orally and given the opportunity to ask questions. Written informed consent were obtained from all participants or their guardians.

### Trial design

This study was an academic prospective, randomized, double-blinded, placebo-controlled trial. Inclusion criteria were at least one episode of vomiting during the last 4 hours and at least one episode of non-bloody diarrhoea during the sickness period. Dehydration was scored as previously described[8] and dehydration more than a moderate degree was an exclusion criterion, see Table 1.

**Table 1:** Exclusion criteria.

**Exclusion criteria**
Severe dehydration
Allergy to ondansetron
Previous abdominal surgery
The use of antiemetics during the last 72 hours
Previous participation in the study
Severe congenital heart defects
Immune deficiency
Malignancy
Malnutrition
Cystic fibrosis
Sickle cell anemia
Fructose intolerance
Diabetes mellitus
Suspected other disease than gastroenteritis

Children who met the inclusion criteria and whose parents or guardians gave written consent to participate in the study were examined by a physician and randomized by a nurse into ondansetron or placebo treatment, blinded as A or B solution. Ondansetron and placebo was given orally. In case of vomiting within 15 minutes, a second dose of the same oral solution was given. Rehydration with oral rehydration solution (ORS) was initiated 15 minutes after ondansetron or placebo according to previous study[8]. ORT was given routinely as a standard treatment at the study hospital, in accordance with WHO recommendations. Hydration with ORS lasted for at least one hour after which the same physician assessed the child again and it was decided whether the child needed intravenous therapy or if the child could return home for further ORT. Stool samples were collected from all participants and examined for enteric viruses and bacteria by real-time PCR.

A blinded member of the study team, located at Linköping University in Sweden, phoned the guardian 24–72 hours after discharge and inquired about the number of vomiting and diarrhoea episodes and at day 7–10, to inquire about the number of days with symptoms (vomiting and/or diarrhoea) after treatment.

### Randomization and blinding

Randomization was carried out by Apotek Produktion & Laboratorier AB (APL), Stockholm, Sweden. Drug and placebo were labelled blinded with A or B and a randomization list was provided. Randomization was done in blocks (n = 8) and the code key was sealed by APL, stored in a locked cabinet at the clinic and remained sealed until the data collection and assessments were fully completed.

### Investigational medical product

Ondansetron, a 5-HT_3_ receptor antagonist in an oral solution (Zofran^®^) of 0.8mg/ml was given in the dose of 0.15mg/kg according to previous studies[8, 24]. Placebo, produced by APL was identical in taste, odour, colour and content to the solution of Zofran^®^ except that it did not contain the active drug.

### Data collection

All data received at the hospital was collected on case report form (CRF) and was kept in a locked cabinet.

The following parameters were recorded at the hospital: fever, weight, hydration level, number of vomiting episodes, number of diarrhoea episodes, tolerance to oral intake, volume of oral intake, need for intravenous rehydration and the need for hospitalization. Guardians received a memory aid to notice the number of vomiting- and diarrhoea episodes the 24 hours after initiation of treatment and they were informed about all the data to be collected at the follow-up by phone contact which was collected at 24–72 hours after treatment and at day 7–10.

### Endpoints

The primary endpoints (E.5.1, EU Clinical Trials Register) was the number of vomiting and diarrhoea episodes during the first 24 hours following treatment. When the study was already ongoing, a secondary endpoint with the number of days with symptoms (diarrhoea and/or vomiting) after treatment was introduced from the 21st participant and forward.

### Viral and bacterial detection by real-time PCR

Rectal swabs (Copan, Brescia, Italy) of 250 μL were mixed with 2 mL of lysis buffer, and this volume was used for extraction of total nucleic acid in an EasyMag instrument (Biomerieux, Marcy l’Étoile, France). The nucleic acids were eluted in 110 μL of eluate buffer and of this volume, 5 μL was used for each real-time PCR reaction.

Real-time PCR was performed in an ABI 7900 384-well system (Applied Biosystems, Foster City, CA) in nine parallel reactions targeting adenovirus, astrovirus, norovirus genotype I (GI) or genotype II (GII), rotavirus, sapovirus, *Campylobacter jejuni*, *Cryptosporidium parvum/hominis*, enterotoxin-producing *Escherichia coli* (ETEC) coding for heat labile toxin (*eltB*) or heat stable toxin (*estA*), enteropathogenic *Escherichia coli* (EPEC) coding for intimin (*eae*) or bundle forming pilus (*bfpA*), *Salmonella*, *Shigella* and *Yersinia*. The sequences of the primers and probe have been previously described[25, 26]. *Clostridium difficile* was detected by using primers (5`-ATCATTACTTCATCTTTGGGGATAGC-3`and 5´-ATCTCGAAGTACAAGTTCATTGTTTACTAA-3) and a probe (CAGGAATTTCAGCAGGTATA) targeting toxin B.

Amplifications were performed in 20 μL reactions containing oligonucleotides and Taqman Fast Virus 1-step Mastermix (Applied Biosystems) for RNA targets or Universal Mastermix (Applied Biosystems) for DNA targets. After a reverse transcription step at 46°C for 30 min followed by 10 min of denaturation at 95°C, 45 cycles of two-step PCR was performed (15 s at 95°C, 60 s at 56°C). In each run, plasmids containing the target regions for all agents were amplified in parallel with patient specimens to verify the performance of each target PCR.

### Sample size calculation and statistical methods

Our assumption was that approximately 60% of the children with AGE would be suffering from a RV- or NoV-infection, a calculation based on previous aetiology studies[4, 27–30].

We estimated the proportion of RV or NoV children who vomited after treatment to be 50% in the control group and 20% in the treatment group. Based on this we calculated that enrollment of 133 children (corresponding to 80 RV or NoV positives) would yield a power of 80%, with two sided significance level of 95%. Due to difficulties in recruiting children, we reached a number of 104 participants during the two-year study period. However, the proportion of children with AGE due to RV and NoV was higher than expected, with 86 children being RV or NoV positive.

The decision to terminate the study was taken by the sponsor, but was due to the fact that the clinic has not the possibility to run the study further.

The Statistical Package for Social Sciences program (IBM SPSS Statistics, version 22.0, Chicago IL, USA) was used for data analysis with significance cut off point of 0.05. The distribution of the continuous endpoint variables deviated significantly from normal distribution (p<0.01 Kolmogorov-Smirnov one-sample test) and were analyzed with Mann-Whitney U-test with two-tailed significance). Proportions were compared using Fisher's exact test with two-tailed significance.

## Results

### Participant flow

A total of 166 patients were screened and 104 patients were enrolled and randomized. Faecal samples were obtained from 102 patients and were analyzed for intestinal pathogens (Table 2).

**Table 2:** Pathogens detected in faecal samples by real-time PCR.

Pathogen	Detected by PCR	% of 102 Participants
Rotavirus	75	73.5
*Clostridium difficile*	19	18.6
NoV GII	13	12.7
Adenovirus 40/41	13	12.7
EPEC *eae*	9	8.8
Sapovirus	9	8.8
Astrovirus	4	3.9
ETEC *eltB*	3	2.9
*Salmonella*	3	2.9
ETEC *estA*	2	2.0
EPEC *bfpA*	2	2.0
NoV GI	2	2.0
*Cryptosporidium*	1	1.0
*Campylobacter*	0	0.0
*Shigella*	0	0.0
*Yersinia*	0	0.0

**Fig 1:**
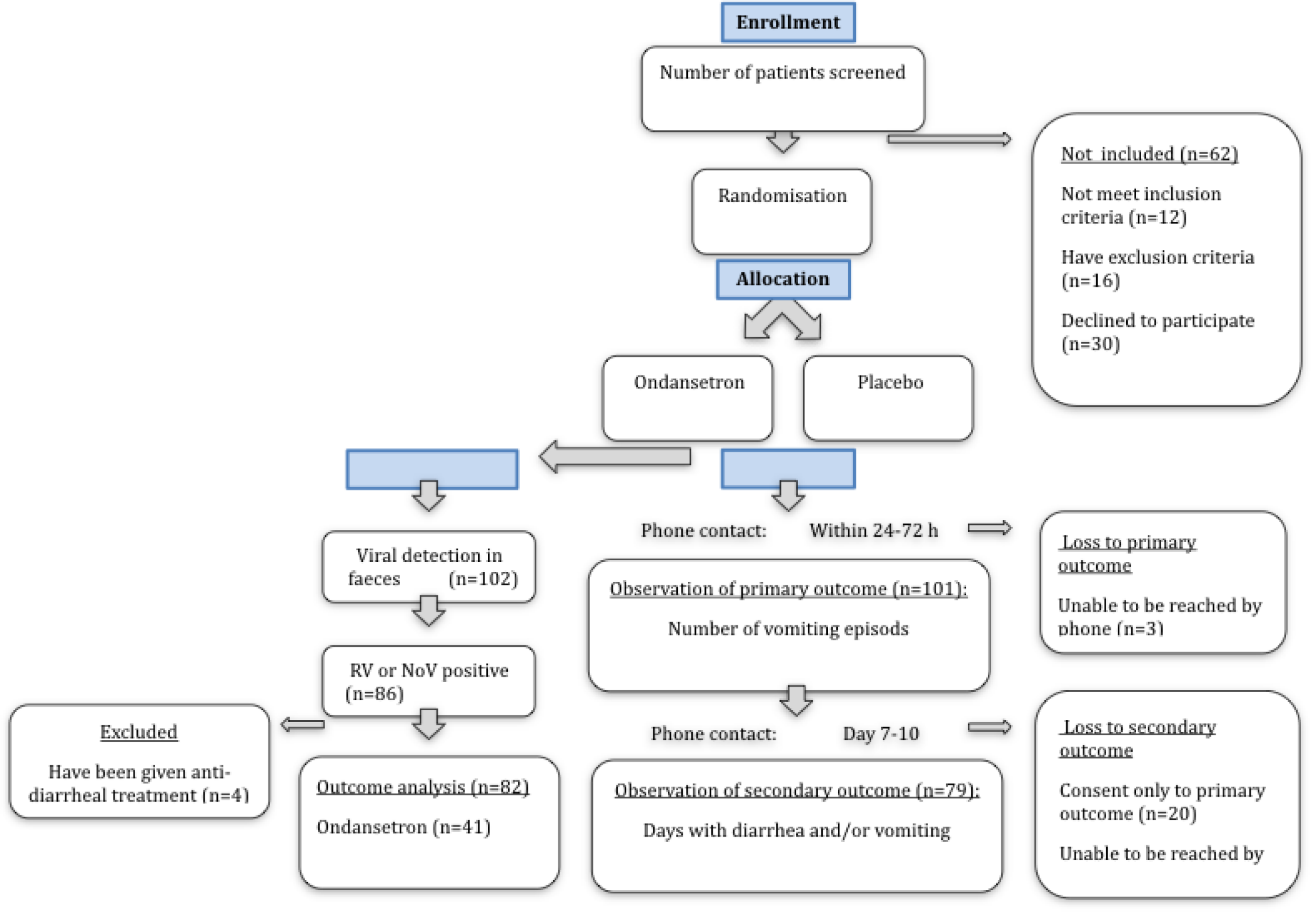
Participant flow chart.

Fig 1 summarizes the flow. Follow-up information of primary endpoints was achieved from 101 children; three children were lost since the guardians could not be reached by telephone. The first 20 included children were only asked for the primary endpoints. The parameter of days with symptoms was added when the study was already ongoing and 79 children completed both primary and secondary endpoint follow-up.

Only children with confirmed RV or NoV infection were included in the outcome analysis. Four children were treated with an anti-diarrhoeal drug, and were therefore excluded from the outcome analysis. Characteristics of the participants with RV or NoV are presented in Table 3. There were no significant differences in the characteristics between the placebo vs. ondansetron treatment groups. The demographics age and gender where 46% respectively 44% males in ondansetron vs. placebo and with age of 27.95 respectively 28.64 months.

**Table 3:** Baseline characteristics of participants in outcome analysis (only children with RV or NoV infection).

Characteristics	Ondansetron group	Placebo group
	n = 40	n = 41
Male–n (%)	19 (47.5)	18 (43.9)
Age–(Months)	28.70 ± 18.38^a^	28.22 ± 22.99^a^
	24.00 ±30.75^b^	24.00 ± 24.00^b^
N of vomiting episodes in preceding 24 hrs	6.41 ± 3.78^a^	7.28 ± 5.67^a^
	5.50 ± 3.69^b^	5.33 ± 3.50^b^
N of diarrhoea episodes in preceding 24 hrs	3.83 ± 2.75^a^	5.38 ± 7.38^a^
	3.00 ± 4.17^b^	3.54 ± 3.00^b^
Days with symptoms before treatment (days)	2.65 ± 1.61^a^	3.07 ± 2.25^a^
	3.00 ± 3.00^b^	3.00 ± 2.50^b^
Rotavirus infection—n (%)	33 (82.5)	34 (82.9)
Norovirus infection—n (%)	7 (17.5)	4 (9.8)
Rotavirus + Norovirus—n (%)	0 (0.0)	3 (7.3)

^a^Mean ± SD

^b^Median ± IQR

### Pathogens

The pathogens detected were mainly viral, especially RV (Table 2). Of the 102 patients analyzed for presence of virus in faeces, 86 children were RV or NoV positive, four of which were positive for both viruses. As shown in Table 2, 75 children (74%) were RV-positive, 13 children were positive for NoV GII (13%) and two children were positive for NoV GI (2%). Of the 102 analyzed participants, 47 children had an infection caused by a single pathogen, two agents were detected in 32 children, three agents were detected in 12 children and four agents were found in one child. In 10 of the faecal samples, none of the screened pathogens was detected.

### Primary endpoints

No difference in the number of vomiting episodes during the first 24 hours after treatment between the ondansetron and placebo groups was observed (p = 0.988; median 0.0 ±2.0 IQR vs. 0.0 ±2.0; Table 4). More than 50% of the children had no vomiting after treatment, independently of ondansetron (21 of 40) or placebo (21 of 41) treatment. There was fewer diarrhoea episodes in RV-infected children receiving ondansetron (p = 0.057; median 1.0 ±6.0 IQR) (n = 33) compared to placebo (median 4.0; ±9.0 IQR) (n = 33) (Table 5), although not statistically significant. A similar observation was made, combining RV- and NoV-infected children (p = 0.063; median 1.0 ±5.0 IQR vs. 3.5 ±9.0; Table 4).

**Table 4:** Outcomes for RV or NoV-infected children.

	Ondansetron group			Placebo group			
Primary endpoint (P) Secondary endpoint (S)	n	Mean (±SD)	Median (±IQR)	n	Mean (±SD)	Median (±IQR)	P-value^a^
(P) No of vomiting episodes after treatment	40	1.43**±**2.29	**0.00±2.00**	41	1.32**±**1.94	**0.00±2.00**	0.988
(P) No of diarrhoea episodes after treatment	40	3.75**±**7.15	**1.00±5.00**	40	5.68**±**8.50	**3.5±9.00**	0.063
(S) Total days with symptoms* during illness	33	4.64**±**2.29	**4.00±3.00**	31	6.10**±**2.87	**6.00±4.00**	0.031

a) Mann-Whitney U-test

*Vomiting and/or diarrhoea

**Table 5:** Outcomes for RV-infected children.

	Ondansetron group			Placebo group			
Primary endpoint (P) Secondary endpoint (S)	n	Mean (±SD)	Median (±IQR)	n	Mean (±SD)	Median	P-value^a^
(P) No of diarrhoea episodes after treatment	33	4.18±7.80	1.00±6.00	33	6.24±9.10	4.00±9.00	0.057
(S) Total days with symptoms* during illness	26	4.69±2.02	4.00±2.00	24	6.25±2.07	6.00±3.00	0.014

a) Mann-Whitney U-test

*Vomiting and/or diarrhoea

### Secondary endpoints

The total days with symptoms in children with RV was a median of 2 days shorter in children receiving ondansetron (p = 0.014; median 4.0; ±2.0 IQR; Table 5) (n = 26) compared to placebo (median 6.0; ±3.0 IQR) (n = 24). Similarly, the days with symptoms were 2 days shorter in ondansetron-treated children with RV or NoV infection (p = 0.031; median 4.0 ±3.0 IQR vs. 3.0 ±4.0; Table 4).

Moreover, the frequency of hospitalization after treatment was lower in the children receiving ondansetron compared to children treated with placebo. Three out of 40 ondansetron-treated children were hospitalized after treatment (7.5%), compared to 8 out of 41 (20%) in the placebo group.

### Significant effect of ondansetron in children with symptoms more than 3 days before treatment

Since only one-dose of ondansetron was given, we wanted to investigate whether the duration of illness had an impact on the effect of treatment. This effect was analyzed by stratification by illness days before treatment (≤ 3 days and >3 days, 3 days being the median). In children with symptoms more than 3 days before treatment, ondansetron significantly reduced diarrhoea in RV- and NoV-infected children during the 24 hours post-treatment period (p = 0.028; median 0.0 ±2.0 IQR vs. 7.0 ±9.0; Table 6). Furthermore, the duration of symptoms was 4½ days shorter in children receiving ondansetron (p = 0.013; median 4.0 ±3.0 IQR; Table 6) compared to placebo (median 8.5; ±3.0 IQR).

**Table 6:** Stratified outcomes of RV or NoV-infected children with symptoms >3 and ≤ 3 days days before treatment.

		Ondansetron group			Placebo group			
Stratified Outcome		n	Mean (±SD)	Median (±IQR)	n	Mean (±SD)	Median (±IQR)	P-value^a^
*Disease duration* No of vomiting episodes after treatment	**>3** **≤ 3**	16 24	0.69±1.58 1.92±2.57	0.00±1.00 1.00±3.00	11 30	0.64±0.81 1.57±2.18	0.00±1.00 0.50±2.00	0.425 0.516
No of diarrhoea episodes after treatment	**>3** **≤ 3**	16 24	2.50±5.38 4.58±8.12	0.00±2.00 2.50±5.00	11 29	5.73±4.74 5.66±9.62	7.00±9.00 3.00±6.00	0.028 0.651
No of days with symptoms* after treatment	**>3** **≤ 3**	12 21	5.17±1.95 4.33±2.46	4.00±3.00 5.00±4.00	8 23	8.50±3.51 5.26±2.12	8.50±3.00 5.00±9.00	0.013 0.191

a) Mann-Whitney U-test

*Vomiting and/or diarrhoea

### Adverse events

Two reports of adverse events were registered, neither of which were considered to be due to the study drug. One of the children got acute otitis media and the other child developed rashes that appeared within 30 minutes after the intake of ORS and disappeared after 60 minutes. The rashes were believed to be associated with the syrup that was used to give a taste to the ORS. Three serious adverse events (SAE) were reported, all of which returned to the hospital within three days after treatment and were dehydrated and hospitalized. Two of the children reported for SAE had been treated with placebo and one child had received ondansetron. All SAE were considered to be due to the natural process of the infection and were reported as unlikely/doubtfully associated with the study drug.

## Discussion

While ondansetron has been extensively studied before in children with gastroenteritis in general, no previous study had aimed at specifically determining whether ondansetron could reduce symptoms in RV- and NoV-infected children.

In this study there was a clear predominance of viral gastroenteritis and RV and NoV were detected in 74% and 14.7% of the children, respectively (Table 2). Viral agents were found in 93% (95/102) of the children and bacterial agents in 31% (32/102).

We found no effect of ondansetron on vomiting 24 hours following treatment, with one dose of 0.15mg/kg. This may be due to the fact that there were very few vomiting episodes in both the ondansetron and placebo groups after treatment, with a median value of zero in both groups, and 51% and 53% of the children reporting no vomiting in the placebo and ondansetron groups, respectively. The few vomiting episodes in both groups thus make it difficult to draw any conclusions regarding whether the drug can limit RV- and NoV-induced vomiting. Rotavirus-infected individuals have a sudden onset of vomiting[27] with a median duration of two days[31]. The comparison of number of diarrhoea and vomiting episodes before and after treatment could not be done because the parents made an assumption about the number of diarrhoea and vomiting before seeking hospital care and they were informed of the study when seeking hospital care and were told to notice and inform the number of vomiting and diarrhoea as well as the number of days with symptoms after the child had received treatment. Since the days with symptoms including vomiting before treatment were a median of 3 days in our study, this may thus explain the limited number of RV-vomiting episodes in ondansetron and placebo groups. Our results are consistent with the study by Ramsook *et al*., who did not find any statistically difference in the number of vomiting episodes and pointed out that it might be because patients enrolled where not sufficiently ill and this might explain why both the ondansetron and placebo group have a median of zero in the follow-up[9].

In our study we found that RV-infected children, treated with ondansetron, had fewer diarrhoea episodes and reduced days with clinical symptoms (p = 0.014; median 4.0; ±2.0 IQR, Table 5) compared to placebo (median 6.0; ±3.0 IQR). The number of NoV-infected children was too few to be analyzed alone (n = 15), thus these were analyzed together with RV-infected children. Similarly, ondansetron-treated children had fewer diarrhoea episodes and reduced days with clinical symptoms (p = 0.031; median 4.0; ±2.0 IQR, Table 4), compared to placebo (median 6.0; ±3.0 IQR). A more pronounced effect on diarrhoea episodes (p = 0.028; median 0.0 ±2.0 IQR vs. 7.0 ±9.0;) and duration of symptoms (p = 0.013; median 4.0 ±3.0 IQR vs. 8.5 ±3.0;) was observed in children with symptoms more than 3 days before treatment (Table 6).

This study is the first to investigate the effect of ondansetron in virus-induced diarrhoea. Previous studies had not taken into account the aetiology and results are from both bacterial and viral caused gastroenteritis. There have been contradictory results published [7–10, 12] and an explanation for these contradictory results may be due to different pathogens, since the pathogenic mechanisms of diarrhoea may not be identical for bacteria and virus. Bacterial infections such as e.g. *Salmonella*, *Shigella* and *Yersinia* are more associated with prolonged and often bloody diarrhoea, a high inflammatory response and less vomiting[27]. In contrast, RV and NoV infections have a clinical picture with a limited inflammatory response[32–38], a relatively short duration and are associated with more extensive vomiting compared to bacterial infections[27, 33, 37, 39]. RV infection has been shown to cause secretory-driven diarrhoea[16, 17] and to involve 5-HT[17, 19], a mediator not only of vomiting, but also intestinal secretion and known to cause diarrhoea[40]. Diarrhoea severity in ondansetron-treated children with AGE has been investigated as an outcome parameter in previous studies[7, 9–12]. The study by Cubeddu and colleagues[12] is the only previous study that has reported the cause of infection[12]. They found that 58% of the included children, aged 6 months to 8 years, suffer from viral infections and 31% from bacteria infections. However, they did not take the pathogens into account in the outcome analysis[12]. They reported more diarrhoea episodes in the 24 hours following ondansetron administration and point out that this may be due to differences in the pathogens, thus participants receiving ondansetron had a greater proportion of bacterial induced gastroenteritis, which may have caused more severe diarrhoea and by a mechanism different from virus[12]. In this study the reduction of diarrhoea episodes was less pronounced in children with bacterial infection (ondansetron; median 1.0 vs. placebo; median 2.5), however, the numbers were too low (n = 30) to make any reliable conclusion.

Our result with reduced number of diarrhoea episodes in the ondansetron treated RV-infected children, is in line with the fact that granisitron[17] and ondansetron can attenuate RV-induced diarrhoea in mice[20] and that constipation is one of the most common side effect by the drug[21, 41]. Furthermore, 5-HT_3_ receptor antagonists are used in the treatment of diarrhoea-dominant IBS[23, 41] and the general effects of 5HT_3_ antagonists implies that they will slow intestinal transit, decrease intestinal secretions and decrease the water content of stool[42]. The half time of ondansetron is age dependent, and in children from 1 month to 12 years between 2.9 to 6.7 hours. A possible explanation for the long time effect of a single dose might be that the recommended and used dose is significantly higher than the dose required to be effective and thus the effect can remain for several days. The effect of ondansetron on diarrhoea episodes was most pronounced in children who have been sick for more than three days before treatment; an effect that might be dose related. It seems that the dose of 0.8mg/kg can reduce or terminate the diarrhoea in the later stage of infection, but seems not effective enough to attenuate diarrhoea in the initial phase of symptoms.

The great advantage with ondansetron is the long safe use and that it can be used in small children. Less diarrhoeal days will result in less viral spread and less days for parents having to stay at home with sick children. The potential benefits of ondansetron treatment to children with viral gastroenteritis including less hospitalization and faster recovery will have a large economical value.

## Limitations

The number of recruited children was lower than the intended target although the study was extended one year. One possible explanation for the difficulty in recruiting children is that usually parents do not take their children to the hospital at an early time-point of gastrointestinal infections and highly dehydrated children could not be included since this was an exclusion criterion for safety reasons. The small number of NoV infected children, 14.7% (15/102), made it difficult to draw any conclusion regarding whether ondansetron had a specific effect on the endpoint parameters in these children, although we believe that the mechanism for RV and NoV are similar due to their similar clinical picture.

## Supporting information

S1 CONSORT Checklist (DOC)

S1 Study protocol (PDF)
