# Supplementary material for: Ondansetron treatment reduces rotavirus symptoms—A randomized double-blinded placebo-controlled trial
Source: PLoS One. 2017 Oct 27;12(10):e0186824. doi: 10.1371/journal.pone.0186824 (PMC5659648; doi:10.1371/journal.pone.0186824)
Supplement: S1 Study protocol — (PDF) [file pone.0186824.s002.pdf]

Study Protocol

Title:

Can 5- HT_3 receptor antagonists be used to limit rota- and norovirus-induced vomiting?

Translated Version 2; Approved by the Ethical Committee in Linköping, Sweden

Table of Contents

SIGNATURES

CONTACTS

LIST OF ABBREVIATIONS USED

SYNOPSIS

BACKGROUND INFORMATION AND RATIONAL

BENEFIT - RISKS

OBJECTIVE

STUDY DESIGN

INCLUSION AND EXCLUSION CRITERIA

THE STUDY DRUG

SIMULTANEOUS ADMINISTRATION OF OTHER DRUGS

REGISTRATION OF POWER

TREATMENTS

REGISTRATION OF SECURITY

STATISTICS

QUALITY CONTROL AND QUALITY ASSURANCE

PROCEDURE FOR RECRUITMENT and obtaining INFORMED CONSENT

HANDLING AND ARCHIVING OF DATA

INSURANCE

REPORTING AND PUBLICATION

REFERENCES

Signatures

Principal investigator :

Birger Trollfors

Sponsor :

Lennart Svensson

Contacts

Lennart Svensson , Professor, Sponsor

molecular Virology

LAB1 Plan 13

581 85 Linköping

lennart.t.svensson @ liu.se

Tel : 010-103 88 03 Fax: 010-103 13 75 , Mobile: 0708-384462

Daniel Novak , MD, PhD . Investigator

Sahlgrenska University Hospital

The Queen Silvia Children 's Hospital

413 45 Gothenburg

daniel.novak @ vgregion.se

Tel : 031-3438072 , Cell: 0708-996253

Birger Trollfors , MD, Professor, Principal Investigator

Sahlgrenska University Hospital

The Queen Silvia Children 's Hospital

41685 Gothenburg

birger.trollfors @ vgregion.se

Tel : 031-3435217 , 070-3680586 Mobile

Marie Hagbom , PhD

molecular Virology

LAB1 Plan 13

581 85 Linköping

marie.hagbom @ liu.se

Tel : 010-103 73 24 , Mobile: 0708-40 14:48

Ove Lundgren , Prof Emeritus

Department of Physiology , University of Gothenburg

Medicinaregatan 11

Box 432

405 30 Gothenburg

ove.lundgren @ fysiologi.gu.se

Tel : 0708-18 75 11

LIST of abbreviations used

AGE = acute gastroenteritis , acute gastrointestinal infection

ORT = reghydration oral therapy , oral rehydration so-called sugar-salt solution

i.v. = intravenous

5-HT = serotonin, 5 - hydroxytryptamine

5-HT3 serotonin = 3 - receptor

NSAID = nonsteroidal anti- inflammatory and analgesic drugs

AE = adverse event , adverse event

SUSAR = Suspected Unexpected Serious Adverse Reaction , suspected serious unexpected adverse reactions

LMV = Drug Administration

REPN = regional ethical review board

GSK = GlaxoSmithKlein

CRF = Case Report Form

ORT = Rehydration

APL = Pharmacy Production & Laboratories AB

Synopsis

The study is planned as a randomized double-blind study in which the effect of ondansetron, with regard to reducing vomiting in children with gastrointestinal infections should be studied. The study is located at the Queen Silvia Children's Hospital in Gothenburg and includes one principal investigater. The purpose of the study is to investigate Whether the Ondansetron (Zofran) (a serotonin receptor antagonist) may reduce vomiting in gastrointestinal infections caused by rotavirus and norovirus (winter vomiting disease). Serotonin Receptor Antagonists are used today against post-operative nausea and vomiting and vomiting caused by chemotherapy treatment, to both children and adults.
Previous studies have been made in e.g. Canada, the US and Turkey, where children with gastrointestinal infections have been treated for vomiting with serotonin receptor antagonists [1-7]. In the US and Canada this drug is today given routineously to children with gastrointestinal infections [8]. This study may provide further use of this class of drugs and if it can reduce vomiting it would facilitate the standard oral treatment, in the form of oral rehydration solution and thereby reduce the number of cases that will need intravenous (i.v.) theraphy. It would be a great economic benefit in terms of reduced time of hospitalization, reduced need for i.v. theraphy and less days for parents to stay home with sick children. To be able to facilitate oral rehydration theraphy (ORT) should save many children's lives in developing countries.

The study will include 215 children aged from 6 months up to 16 years, and will last from spring 2013 until the number of 215 children have been reached, which means that it may continue in 2014.
Children who seek care at the Queen Silvia Children's Hospital for acute gastrointestinal infection is assessed by a physician and if they meet the inclusion criteria and agree to participate in the study, they will be randomized to receive Ondansetron treatment or serve as controls (receive placebo).

Background information and Rational

Intestinal infections are at the third place among all causes of disease burden and is responsible for 1.7 to 2.5 million deaths per year [10]. Acute intestinal infections, acute gastroenteritis (AGE) are among the most common diseases and the mortality is highest among children and the elderly[11]. Recent studies have identified human calicivirus (eg, norovirus) as the most common cause of AGE in humans [10] and rotavirus is the most common cause of infantile gastroenteriter in the world, accounting for about a third of the severe cases of AGE required hospitalization [12]. More than 600,000 children die annually because of rotavirus infection, most of which occur in developing countries [13].
First-line treatment of AGE focuses on recovering the fluid balance and give the children fluids to restore the electrolyte balance. Oral rehydration solution (ORT) containing glucose and electrolytes, leads to rehydration, and is prescribed for mild to moderate dehydration, while i.v. administration of fluid is recommended in severe cases [14]. ORT is still underutilized in many countries (eg developing countries) [6], especially in children with extensive vomiting, where pediatricians are more likely to give i.v. theraphy instead of ORT [1]. Antiemetic drugs such as granisetron and ondansetron (serotonin 3 (5-hydroxytryptamine-3, 5-HT 3) receptor antagosnists), has

been shown to have a good antiemetic effect after chemotherapy or in situations with vomiting after surgery [15 , 16]. Furthermore, Ondansetron was shown to be effective to facilitate ORT in children with AGE in seven independent clinical studies [1-6,14]. A systematic review [17] shows that Ondansetron decreases the risk of persistent vomiting, reduces the use of i.v treatment and hospital admissions in children with vomiting. Although Ondansetron has been shown to have significant effect on the reduction of vomiting during AGE, there is no information available if vomiting due to rotavirus or norovirus can be attenuated with a 5-HT 3 receptor antagonist. We have in an animal model demonstrated that rotavirus diarrhea could be attenuated by a 5-HT3 receptor antagonist [18], and that rotavirus infection cause activation of brain areas of the vomiting center [19], which together indicate a common triggering mechanism for fluid loss and vomiting during rotavirus and probably also norovirus infection. Our hypothesis is that rotavirus and norovirus stimulate the release of 5-HT from sensory cells in the intestine and through nerve stimulation give rise to diarrhea and vomiting.

This study aims to facilitate oral fluid therapy for rotavirus- and norovirus-gastroenteritis, by reducing vomiting and thus enhance the oral intake of fluid.

Benefit-Risk Assessment

This study aims to facilitate oral fluid therapy for gastrointestinal infections caused by rotavirus and norovirus, by reducing the vomiting. It would be a great economic benefit in terms of reduced risk for viral transmission, reduced hospitalization time, reduced i.v. treatment and reduced number of days for parents to be home with sick children. The ability to facilitate ORT would save many children's lives in developing countries.

<u>Risk Assessment</u>

a). Risks related to study drug:

This is an already established drug on the market and used for both children (from 6 months of age, in the doses to be used) and adults, for treating vomiting caused by chemotherapy or post surgery. Several studies with oral administration of Ondansetron, for treatment of nausea and vomiting caused by chemotherapy has been performed. Ondansetron has been given to child from 6 months and older.

Overdose: There is little information on overdose of ondansetron (no information on www.giftinfo.se) but 48 mg oral intake in adults gave a mild intoxication. There is no antidote for Ondansetron, the treatment is symptomatic. The risk of overdose is considered very low when the drug is dosed and administered by the research nurse.

Side effects: The most serious adverse reaction is with i.v. treatment with Ondansetron. According to the SPC, the most common side effects per os administration is headache, constipation and experience of flushing and heat sensation. These can be treated symptomatically. Serious, life-threatening side effects are rare or very rare, and are: immediate hypersensitivity reactions, sometimes including anaphylaxis. This is noted during the observation period in the emergency room when the child is given ORT in accordance with customary routines. Cardiac arrhythmias (incl. Torsade de pointes) and hypotension can be observed in the second assessment of the dehydration score.

Contraindications:
The investigational medicinal product contains sorbitol. Patients with intolerance to fructose should not take ondansetron.
A well made systematic Cochrane review from 2006 compared ORT with i.v therapy regardless of the dehydration score (26). 17 studies were included, 9 were from high-income countries. ORT was associated with a 4% increased risk of failure rehydration but if you did about the meta-analysis with a uniform definition of failure rehydration, there was no difference between failure rate of ORT compared to the i.v. therapy. The i.v. therapy, however, was associated with a higher risk of phlebitis (inflammation of the artery walls).

b) Related medical devices:
Ondansetron is administered with a syringe (no needle) against the inside of the cheek by an experienced pediatric nurse (research nurse). All per oral medicine in children emergency room is given in this way and has not caused any risks.

c) Study Specific inquiries:
There are no invasive (ie cannulation or blood sampling) measures in the study that goes beyond the regular medical care. Should it be required to take blood or needed an i.v. needle, this is based on a medical assessment independent of the study. The only sample that is taken is a stool samples from the diaper, potty or toilet. If no stool is avaiable, a feaces sample is taken by an experienced pediatric nurse (research nurse) from the rectum (rectal swab). This is done regularly of children at the children's emergency room and this is not considered an invasion of privacy, or cause to any risks.

Benefits valuation
The risks is seen as minimal, as only one dose will be given to these children (or 2 doses if the child vomits within 15 minutes of drug / placebo administration). The ability to reduce vomiting and thus facilitate oral rehydration will favour the children to recover from dehydration very quickly and thus the child will be able to accompany the guardians home for further treatment with oral rehydration. Unlike from Sweden, where the established drug is not used for vomiting caused by gastrointestinal infections, it is used routinely for gastrointestinal infections in countries such as the USA and Canada (8).
In summary, the benefits outweigh the discomfort and the risks of ingestion of Ondansetron in this study.

Objective

The primary issue is to study whether the serotonin receptor antagonist ondansetron can reduce vomiting in rotavirus and norovirus infection.
The primary endpoint is the number of vomiting. Emetic episodes less than 2 minutes between is counted as one vomiting. Non-productive retching, drooling, or ejection of oral fluid is not considered a vomiting.
The secondary issue is whether there is a difference between the placebo group and the Ondansetron group in the number of diarrhoea episodes (gas passage with the exchange does not count as an diarrhoea episod).

Study Design

The study is a prospective, randomized, double-blinded study, with therapeutic use of an already established drug, an explorative phase II study, a new indication for the use of ondansetron. Placebo will be included.
Children between 6 months and 16 years, with vomiting and diarrhea will be recruited in the children emergency room at Queen Silvia Children's Hospital and are requested to participate in the study with the consent of guardians. Children will be recruited during the gastroenteritis season 2013 and if necessary, during 2014, until the number of children have been met (215 children). All patients will be examined by a physician. Children who have been vomiting (without blood, bile or feces) at least one time in the last four hours and had at least one non-bloody diarrhea during the sickness period, and which has at most a mild to moderate dehydration can be included in the study. Dehydration is scored according to previous study [2].

Physicians, as delegated by the principal investigator, will give both oral and written information to the child and its guardians, regarding what a possible participation in the study involves. Guardians and the child will have the opportunity to ask questions. They are also informed that the participation is entirely voluntary and that they could at any time choose to discontinue the study. In the information it is clear that participation or non-participation do not affect the child's treatment or hospital stay.

Children who meet the inclusion criteria and provide written consent (the parents) to participate in the study will be randomized to receive ondansetron treatment or serve as controls (receive placebo). The treatment with Ondansetron (Zofran), oral solution 0.8mg/ml in the dose 0.15mg /kg according to previous studies [8,9], or placebo will be administered by a nurse, in randomized order. Guardians and medical staff will be unaware of what the child was given, placebo or ondansetron. Rehydration with ORT is initiated 15 minutes after treatment with either ondansetron or placebo, according to previous study (Freedman et al 2006). ORT is given as standard treatment, in accordance with WHO recommendations.

(http://www.who.int/whosis/whostat2006Under5WithDiarrhoeaReceivedORT.pdf).

Hydration with ORT will last for an hour after which the child is assessed by the same physician and it is assesses whether the child needs i.v drip or if the child can go home for further ORT, in accordance with the previous study [2]. In order to monitor the patient's health status, the following parameters will be recorded: fever, weight, hydration level, number of vomiting episodes, number of diarrhoea episodes, tolerance of oral intake, the volume of oral intake, possible need for i.v. rehydration and need for hospitalization [2].
If the patient assimilates ORT treatment and can go home, a person from the study team will make a phone call to the guardians 24-72 hours after they have went home, asking for information, such as: the general state of the child in the home, number diarrhoea episodes, number of vomiting episodes and possible side effects of the drug. In cases where the child is not urinating over 8 hours or get worsen general health, this can indicate too little fluid intake and the child is asked again seek children's emergency room. If a patient is asked to come back to the hospital, he/she will be assessed by a physician for i.v. drip treatment and possible hospitalization. If this

assessment is done by a doctor who does not participate in the study, information about the patients will be given to the principal investigator of the study. The children emergency room is open 24 hours a day and all employees will be familiar with the study.

Stool samples collected from all subjects and these will be examined for enteric viruses by ELISA test for rotavirus and norovirus. After a positive confirmation of rotavirus or norovirus so characterized the strains by genotyping as previously described [20,21]. The viral diagnosis will be performed by Professor Lennart Svensson's lab in the Department of Molecular Virology in Linköping and at the microbiology laboratory at Sahlgrenska hospital. The fecal samples will be stored in a biobank at Linköping University.

Inclusion and Exclusion Criteria

215 children from 6 months to 16 years, with symptoms of gastrointestinal infection, such as diarrhea and vomiting, will be recruited at the children emergency room at the Queen Silvia Children's Hospital. Patients receive information and request to participate in the study with the consent of guardians. All patients will be examined by a physician.

Inclusion criteria are: Children who have been vomiting (non-blood, non bile and not feces) at least once in the last four hours and had at least one non-bloody diarrhea during the period of illness, and as most have a mild to moderate dehydration.

Exclusion criteria are: severe dehydration or other diseases that can mask the assessment of dehydreringsgraden (renal failure or hypoabluminemi), known allergy to Ondansetron, previous abdominal surgery, the use of antiemetics during the last 72 hours, and previous participation in the study. Other exclusion criteria are severe congenital heart defects, immune deficiency, malignancy, malnutrition, cystic fibrosis, sickle cell anemia, fructose intolerance, diabetes mellitus and suggestive of a disease other than gastroenteritis on physical examination (such as focal neurological signs or symptoms of increased intracranial pressure, acute abdominal surgical affliction and supraventricular tachycardia).
All children with gastroenteritis regardless of whether they participate in the study or not, will have the same information, to come back to the emergency room if the child is not urinating in time of 8 hours or get worse general health. A new medical assessment by a physician will be done prior to any. i.v. drip and/or hospitalization with i.v drip. Although i.v. drip is given or if hospitalization is needed, the child will not be excluded from the study.
The participant can at any time choose to cancel their participation in the trial. Doctors/sponsor may at any time terminate the study due to side effects or that the patient does not follow the procedures in the study protocol.

In the study, we will use a dehydreringsscore consisting of seven different parameters, as Freedman et al have used (2). All parameters will be evaluated in children under 24 months while children over 24 months are evaluated in six parameters (the ability to produce tears is not evaluated). The dehydration score have been discussed and tested

in cooperation between investigater and principal investigator. Urine output (quantity) and color are reported by guardians.

High values indicate more dehydration. Children under 24 months with scores between 10-17 and older children with a score between 8-15 is estimated to have normal to moderate dehydration. Children under 24 months with 18 or more points and older children with 16 or more points is considered to be severely dehydrated. Severe dehydration is an exclusion. All investigators have been trained in the scoring.

It is extremely important to not miss a hypertonic dehydration but it is also important to make as few invasive procedures on the children as possible. In the children emergency room, we follow clear guidelines that are based on the most recent NICE guidelines for the diagnosis, assessment and treatment of gastroenteritis in children (23). According to these guidelines, it is not justified to do regular sampling of all children. Sampling should be done only when the i.v. therapy should be started or if there are symptoms or signs of hypertonic dehydration. Hypertonic dehydration should be suspected if the child has jerky movements, increased tone, hyperreflexia, convulsions, or reduced consciousness. Other symptoms are imperative thirst and fast weight loss (24). Hypertonic dehydration happens most often in children under 6 months and is rare after the age of 2 years (23).
In children emergency room, and in the planned study, we do as follows: In severe dehydration, the child should be hospitalized and needle should be set as well as check of acid-base status, including electrolytes. These children are not included in the study. In a child with moderate or severe dehydration who show age or symptoms of hypertonic dehydration, we take a capillary Na. S-Na above 150mmol /l is considered as hypertonic dehydration and are treated (24). If the S-Na is between 145mmol /l and 150 mmol /l, we will call kemlab that complement existing blood with the S-CI. S-Na of 145 mmol /l in combination with the S-Cl of 115mmol /l is considered hypertonic dehydration (24). Children with hypertonic dehydration is hospitalized. NICE recommend ORT treatment before i.v. treatment unless i.v. treatment is necessary due to severe dehydration.

The Study Drug

Ondansetron is a serotonin receptor antagonist used to reduce nausea and vomiting after surgery, chemotherapy or radiation therapy. We now want to investigate if ondansetron can attenuate vomiting caused by gastrointestinal infection, whether it can specifically reduce vomiting during rotavirus and norovirus infection. Zofran, an oral solution of 0,8mg /mL at an dose of 0.15mg /kg will be used, according to previous studies [8,9].
The drug Zofran is manufactured by GlaxoSmithKline (GSK) and are available from the pharmacy. The drug will be re-labeled by the APL (Pharmacy Production & Laboratories AB), so that the person who gives the drug not are able to see if it is the active drug or the placebo. APL will deliver both the drug and placebo to the hospital pharmacy, at Östra Hospital, Gothenburg. The medicine will be kept at the children emergency room inaccessible to others except research nurses and the principal investigator, separate from regular medicine. The medicine is given by a research nurse at the children's clinic during the day or at night by a nurse who received the

delegation of the lead investigator. The nurse checks that the patient ingests the drug / placebo. Children who vomit within 15 minutes from medication gets a new dose according to previous study [2].

Simultaneous Administration of Other Drugs

No other drugs will be used simultaneously. Analgesic and antipyretic drugs such as NSAIDs (nonsteroidal anti-inflammatory and analgesic drugs) and paracetamol should be avoided if possible. In the case of NSAIDs or acetaminophen to be given (eg high fever), it must be documented in the CRF.
If the participant is taking other medications for any reason, this will be recorded in the documentation and you have to consider whether the parcitipant is fit to participate in the study or not. Use of antiemetics during the last 72 hours are exclusion criteria for the study.

Registration of Effect

The following parameters will be assessed in the children emergency room: Change in dehydration score after placebo/ondansetron followed by ORT, number of vomiting episodes, number of diarrhoea episodes, tolerance of ORT, the volume of ORT, need of i.v. rehydration and needs of hospitalization.
The patient will be phoned 24-72 hours after returning home, by a person from the research team, and asked about information about the general condition, number of diarrhoea and vomiting episodes and possible side effects of the drug.
Stool samples will be collected from the participants of the study. The sample is taken with a small spoon (about 5 ml volume). From small children sample taken from the diaper. If it's just fluid in the diaper this can this be pushed out with a spoon. Stool samples will be routinely analyzed at the microbiological laboratory at Sahlgrenska Hospital and then sent to the Department of Molecular Virology at Linköping where characterization of rotavirus and norovirus will be performed [19, 20].

Treatments

Ondansetron
Zofran (0,8mg /mL) oral solution. See information under the heading "investigational medicinal product"

Placebo
The placebo solution as study drug Zofran but without the active substance. Placebo are produced, packaged and labeled by APL.

ORT
Oral rehydration solution, containing glucose and salts, which leads to rehydration, which is prescribed for mild to moderate dehydration, while i.v. supplying liquid is recommended in severe cases [14]. Oral rehydration therapy (ORT) is mixed by the responsible nurse. 1 liter of water + ½ teaspoon salt + 6 teaspoons of sugar (as recommended by healthcare advice). Guardians are instructed to provide the liquid in small amounts, teaspoon, every 3 to 5 minutes. Max intake orally is 12.5 ml/kg/hour.

Registration of Security

When a child arrive to the emergency room, a triage nurse will asess the child. The patient is assessed for vital signs by “metts-Pediatric” (metts p v1.0). “Medical Emergency Triage and Treatment System” is an internationally developed tool for initial and secondary assessment of children with good reliability (25). Vital parameters included are airway, breathing rate, pulse, consciousness, temperature and capillary refill. In addition to initial and secondary (after ORT) assessment by the physichian, the child is regularly checked by metts p vital signs throughout their stay. These parameters are checked by the research nurse and registered.

Undesirable event - 'Adverse Event (AE) "
Adverse events are all adverse events occurring during the study, regardless of whether they are related to the study drug or not. All these are assessed by the principal investigator and recorded in the investigator's brochure. Concurrent illnesses reported at baseline will not be reported as "undesirable events" (AEs). However, a worsening of the patient's disease during the study should be assessed as an AE SAE options depending on what happened. The patient can always visit the emergency room for AEs, and the emergency room is open all hours and all staff are familiar with the study. Principal investigator (or other investigators delegated by the principal investigator) will be available 24 hours a day on the phone.

The severity is judged by the following:

Mild AE = AE tolerated easily:
Side effects of the drug such as headache, feeling hot, dizziness, fatigue, hiccups, and coughs. Adverse events in previous studies show that 90% had a feeling of fatigue and a few got a cough (27).

Moderate AE = AE which affect the ability of the patient to normal activity and provides significant discomfort:
Mild to moderate dehydration, transient. Constipation.

Severe AE = an AE which largely affects the ability of the patient to normal activity and causing great discomfort.
For serious AE, the relationship with the study drug should be assessed ('not related', 'possibly related' or 'probably related'). AEs judged to be related to study drug will be followed up by the principal investigator to allow assessment of reversibility.
Serious Adverse Event (SAE)
An important medical event, regardless of dose, requiring medical or surgical intervention to prevent serious outcomes (death, life-threatening situation, hospitalization, significant disability or incapacity). This shall be reported by the investigator within 24 hours.
For each SAE, the relationship with the study drug will be assessed and followed up by the principal investigator to allow assessment of reversibility.

SUSAR reporting ("Suspected Unexpected Serious Adverse Reaction"
Side effects that occur and which are not included in the SPC in FASS will be reported by lead investigator as follows:

A suspected unexpected serious response reaction to the study drug (SUSARs) that are fatal or life-threatening should be reported to the Medical Products Agency (MPA) and the Local Ethical Committee (REPN) within 7 days of date from that the principal investigator became aware of the incident. Completion of the report should be sent to the authorities within a maximum of 15 days.
A suspected unexpected serious response reaction to the study drug (SUSARs) that are not fatal or life-threatening shoul be reported to LMV and REPN within 15 days from the date that the principal investigator became aware of the incident. Completion of the report should be sent to the authorities as soon as possible.
When SUSARs occurring in Sweden in an investigator-initiated study of non-commercial sponsor, who is not able to directly report to the European database on adverse effects (the so-called testing module of EudraVigilance), a CIOMS form (available at the NLS website) can be used. This form is sent to registrator@mpa.se.

Statistics

We will at the time when the children receiving placebo/Ondansetron not be aware of the etiology of children's gastroenteritis. However, the study will be carried out during the high season for viral gastroenteritis. 215 children is the number that will be included and we believe we have sufficient number of children to be able to do statistical calculations. We have no similar studies to relate to, but with previously published data, we estimate that more than 60% of the acute gastroenteritis will be caused by rotavirus (Uhnoo and Svensson, Journal of Clinical Microbiology, Mar. 1986, p. 551- 555; Uhnoo et al, J Infect. 1986 Christmas; 13 (1): 73-89.) and norovirus (CDC), and we believe therefore that 129 children (60%) are sufficient numbers to see a statistical difference. In fact, this study is one of the largest (along with Freedman et al, N Engl J Med 2006; 354: 1698-1705April 20, 2006, which included 215 children) of the studies whatsoever made by Ondansetron and gastroenteriter on children.

Quality Control and Quality Assurance

Before any study related action/investigation can begin, the patient and their guardians are informed and signed the consent, which should also include that be a monitor and any inspection will have access to patient records, to verify the source data. The head of the department must give their permission for the monitor to have access to relevant patient data in patient records. A confidentiality agreement should be maintained between the head of the department, who is responsible for patient records and the monitor.
Monitoring of the study will be conducted by an independent monitor from Linköping Academic Research Center (LARC).

The monitor will ensure that:

- Compliance with the Protocol
- Correct data will be collected for the study, in "Case Report Form" -CRF
- The adverse events are reported as LVFS 2011: 19
- Handling of the drug is made under the Protocol and regulations for clinical trials.
- Original data and study related documents are available and assessible and that all documentation is saved/archived.

Principal investigator shall ensure that the monitor has access to CRF, patient and original laboratory data, to check the source data relevant to the study, without compromising patient privacy. Principal investigator is also aware that the inspection of the authority might be implemented.

Procedure for Recruitment and Obtaining Informed Consent

Consent process will to be carried out in accordance with ICH GCP guidelines and the Helsinki declaration. The guardian (as these subjects are under 18 years) must provide written consent alternative oral consent on the phone (and in retrospect one signature per guardian) to participate in the study, before any study related action can be performed. A copy of the patient information and of the signed consent will be given to the guardian.
A physician will give both oral and written information to the child and its guardians, regarding what a participation in the study means. Guardian and the child will have the opportunity to ask questions. They are also informed that participation is entirely voluntary and that they could at any time decide to terminate the study without medical care deteriorates.
The guardian's signature gives consent to participate in the study, agree monitoring and pharmaceutical authorities to get full access to medical records, etc. (the privacy).
Inclusion of patients in the study will start once the written approval of the Regional Ethical Review Board (REPN) as well as authorization from the Medical Products Agency (MPA) is available.

Operation and archiving of data

Patients is identified with his initials and a serial number. The patients' full identity linked to the serial number of the study will be made on a patient identifier list stored in a locked cabinet.
Patient data will be processed and handled by the principal investigator at the Queen Silvia Children's Hospital, Gothenburg.
Principal investigator has a binder with relevant content for the study as patient forms, patient identification list, the original patient information and obtained consent for the study. This binder will be kept away from unauthorized, but the responsible for the study will have full access.
Study document and source data will be archived for at least 10 years after the study report has been written and submitted to the MPA.

Insurance

Patients are insured by the Pharmaceutical Insurance and Patient Injury Act.

Reporting and Publishing

Study data are owned by the sponsor.
Processing of the results will be made as soon as the study is completed, when 215 children have been enrolled in the study. Results from the study will be published in international journals.

References

1. Yilmaz HL, Yildizdas RD, Sertdemir Y (2010) Clinical trial: oral ondansetron for reducing vomiting secondary to acute gastroenteritis in children--a double-blind randomized study. Aliment Pharmacol Ther 31: 82-91.
2. Freedman SB, Adler M, Seshadri R, Powell EC (2006) Oral ondansetron for gastroenteritis in a pediatric emergency department. N Engl J Med 354: 1698-1705.
3. Roslund G, Hepps TS, McQuillen KK (2008) The role of oral ondansetron in children with vomiting as a result of acute gastritis/gastroenteritis who have failed oral rehydration therapy: a randomized controlled trial. Annals of emergency medicine 52: 22-29 e26.
4. Ramsook C, Sahagun-Carreon I, Kozinetz CA, Moro-Sutherland D (2002) A randomized clinical trial comparing oral ondansetron with placebo in children with vomiting from acute gastroenteritis. Annals of emergency medicine 39: 397-403.
5. Cubeddu LX, Trujillo LM, Talmaciu I, Gonzalez V, Guariguata J, et al. (1997) Antiemetic activity of ondansetron in acute gastroenteritis. Alimentary pharmacology & therapeutics 11: 185-191.
6. Stork CM, Brown KM, Reilly TH, Secreti L, Brown LH (2006) Emergency department treatment of viral gastritis using intravenous ondansetron or dexamethasone in children. Academic emergency medicine : official journal of the Society for Academic Emergency Medicine 13: 1027-1033.
7. Pfeil N, Uhlig U, Kostev K, Carius R, Schroder H, et al. (2008) Antiemetic medications in children with presumed infectious gastroenteritis--pharmacoepidemiology in Europe and Northern America. The Journal of pediatrics 153: 659-662, 662 e651-653.
8. Freedman SB, Sivabalasundaram V, Bohn V, Powell EC, Johnson DW, et al. (2010) The Treatment of Pediatric Gastroenteritis: A Comparative Analysis of Pediatric Emergency Physicians' Practice Patterns. Acad Emerg Med.
9. Marchetti F, Maestro A, Rovere F, Zanon D, Arrighini A, et al. (2011) Oral ondansetron versus domperidone for symptomatic treatment of vomiting during acute gastroenteritis in children: multicentre randomized controlled trial. BMC pediatrics 11: 15.
10. Girard MP, Steele D, Chaignat CL, Kieny MP (2006) A review of vaccine research and development: human enteric infections. Vaccine 24: 2732-2750.
11. Glass RI, Bresee J, Jiang B, Gentsch J, Ando T, et al. (2001) Gastroenteritis viruses: an overview. Novartis Foundation symposium 238: 5-19; discussion 19-25.
12. Rodrigo C, Salman N, Tatochenko V, Meszner Z, Giaquinto C (2010) Recommendations for rotavirus vaccination: A worldwide perspective. Vaccine 28: 5100-5108.
13. Parashar UD, Gibson CJ, Bresse JS, Glass RI (2006) Rotavirus and severe childhood diarrhea. Emerg Infect Dis 12: 304-306.
14. Reeves JJ, Shannon MW, Fleisher GR (2002) Ondansetron decreases vomiting associated with acute gastroenteritis: a randomized, controlled trial. Pediatrics 109: e62.

15. Patel RI, Davis PJ, Orr RJ, Ferrari LR, Rimar S, et al. (1997) Single-dose ondansetron prevents postoperative vomiting in pediatric outpatients. Anesthesia and analgesia 85: 538-545.
16. Schwella N, Konig V, Schwerdtfeger R, Schmidt-Wolf I, Schmid H, et al. (1994) Ondansetron for efficient emesis control during total body irradiation. Bone marrow transplantation 13: 169-171.
17. DeCamp LR, Byerley JS, Doshi N, Steiner MJ (2008) Use of antiemetic agents in acute gastroenteritis: a systematic review and meta-analysis. Arch Pediatr Adolesc Med 162: 858-865.
18. Kordasti S, Sjovall H, Lundgren O, Svensson L (2004) Serotonin and vasoactive intestinal peptide antagonists attenuate rotavirus diarrhoea. Gut 53: 952-957.
19. Hagbom M, Istrate C, Engblom D, Karlsson T, Rodriguez-Diaz J, et al. (2011) Rotavirus stimulates release of serotonin (5-HT) from human enterochromaffin cells and activates brain structures involved in nausea and vomiting. PLoS pathogens 7: e1002115.
20. Nordgren J, Bucardo F, Dienus O, Svensson L, Lindgren PE (2008) Novel light-upon-extension real-time PCR assays for detection and quantification of genogroup I and II noroviruses in clinical specimens. Journal of clinical microbiology 46: 164-170.
21. Nordgren J, Bucardo F, Svensson L, Lindgren PE (2010) Novel light-upon-extension real-time PCR assay for simultaneous detection, quantification, and genogrouping of group A rotavirus. Journal of clinical microbiology 48: 1859-1865.
22. Fedorowicz Z, Alhashimi D, Alhashimi H (2007) Meta-analysis: ondansetron for vomiting in acute gastroenteritis in children. Alimentary pharmacology & therapeutics 26: 1086; author reply 1087.

Attachments

- Flowchart
- Labeling Proposal and pharmaceutical documentation of placebo and Zofran
- Consent Form
- User's handbook (Investigarors Brochure)
- Written patient information
- CV for researchers with primary responsibility
